# Supplementary figures and images for: Level-Dependent Subcortical Electroencephalography Responses to Continuous Speech
Source: eNeuro. 2024 Aug 23;11(8):ENEURO.0135-24.2024. doi: 10.1523/ENEURO.0135-24.2024 (PMC11351020; doi:10.1523/ENEURO.0135-24.2024)

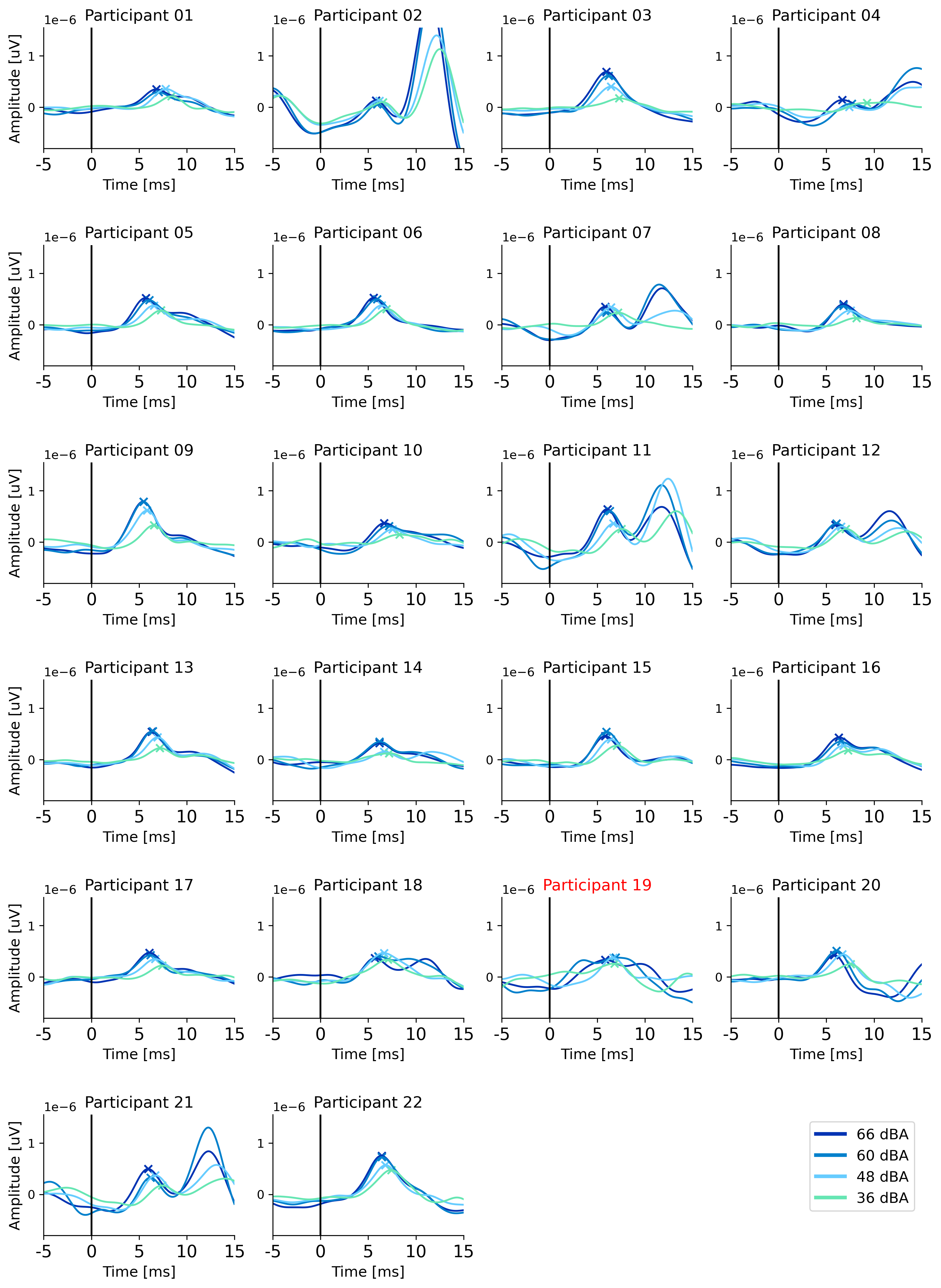

Supplement: Figure 1-1 — Individual click ERPs. All the click ERPs for each participant 694 calculated on the full dataset are shown. Some participants also have a large post-auricular muscle reflex or middle latency responses. The peak in the range of 4-10 ms is automatically detected as the wave V peak (marked with an X in the plots). Participant 19 was rejected and not analyzed due to the lack of clear click ERPs or speech TRFs, but is shown here for completeness. Download Figure 1-1, TIF file. [file eneuro-11-ENEURO.0135-24.2024-s001.tif]

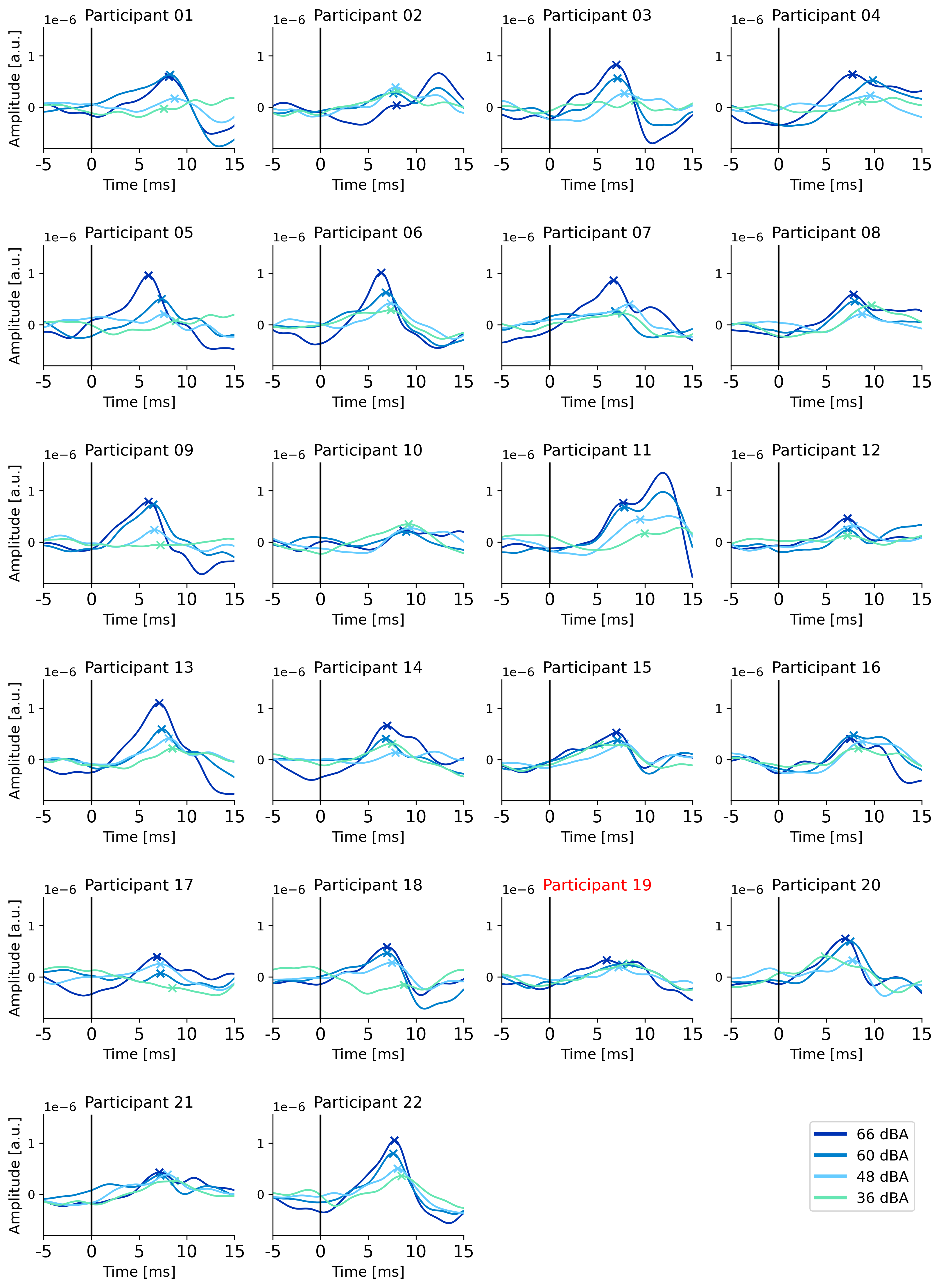

Supplement: Figure 1-2 — Individual Speech TRFs: RS Predictor. RS TRFs for each participant calculated on the full dataset are shown. Note that TRFs are quite noisy and have broad peaks. Download Figure 1-2, TIF file. [file eneuro-11-ENEURO.0135-24.2024-s002.tif]

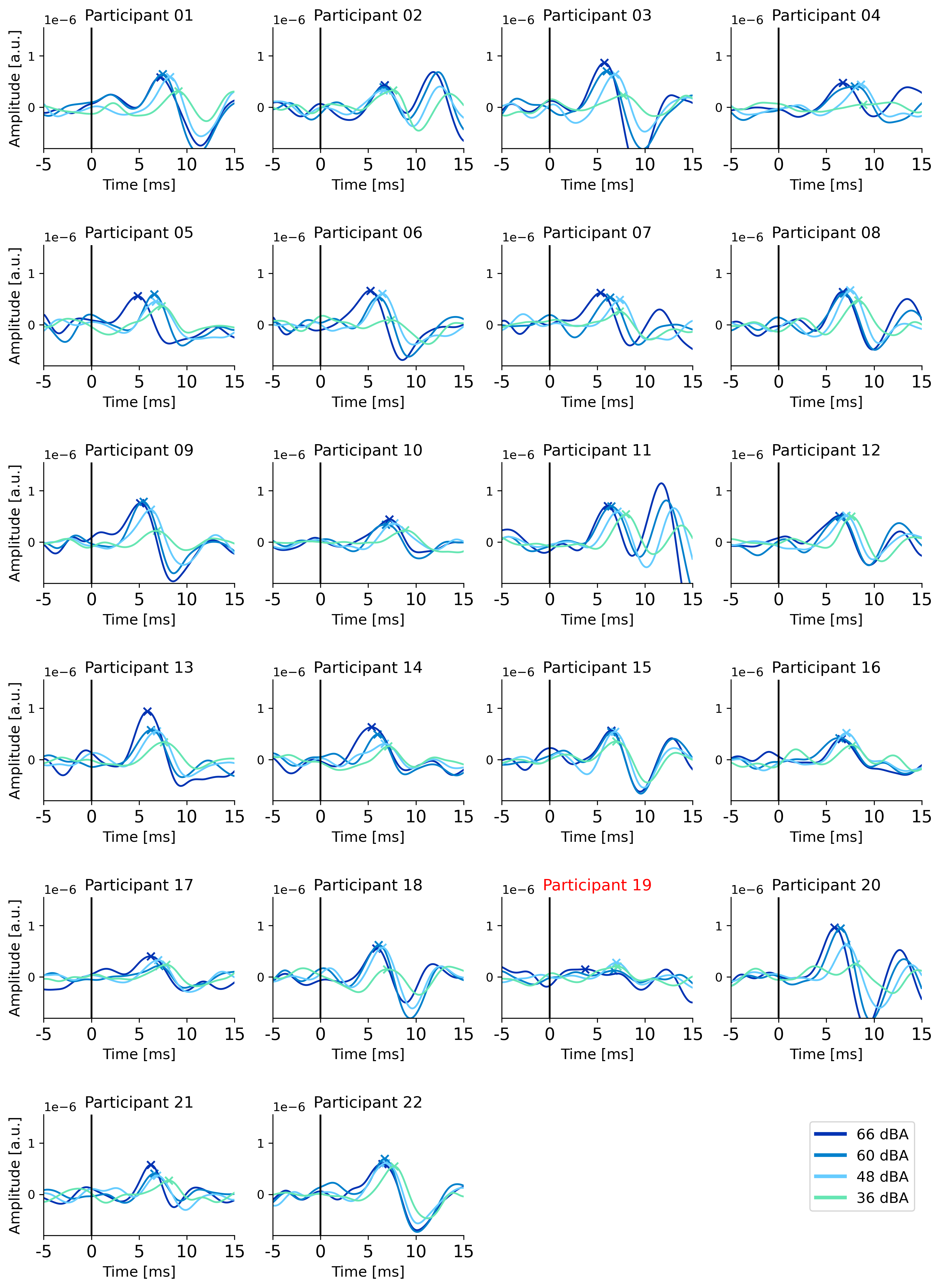

Supplement: Figure 1-3 — Individual Speech TRFs: GT Predictor. GT TRFs for each participant calculated on the full dataset are shown. Note that both level-dependent amplitude and latency effects can be seen for most participants. Download Figure 1-3, TIF file. [file eneuro-11-ENEURO.0135-24.2024-s003.tif]

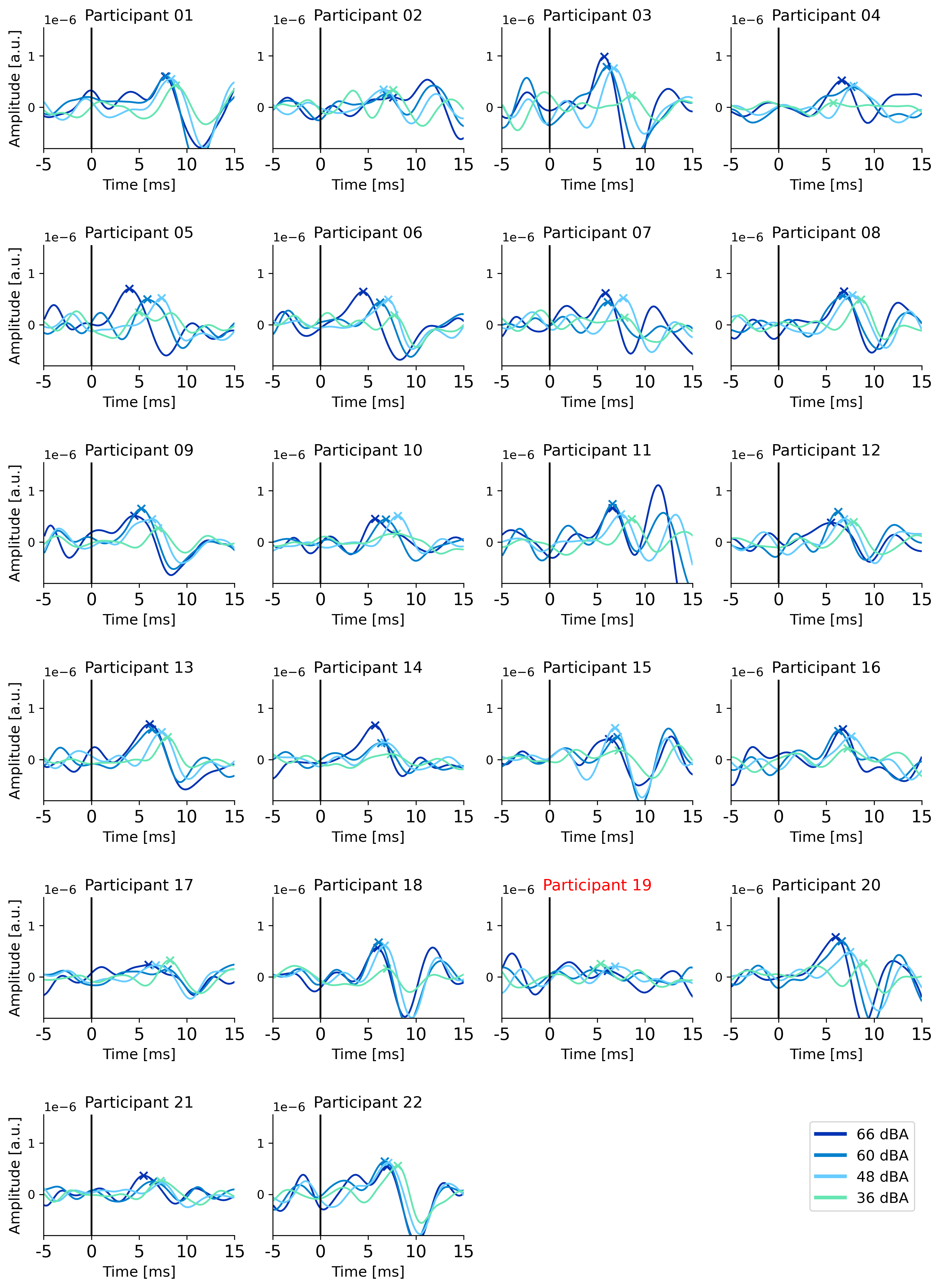

Supplement: Figure 1-4 — Individual Speech TRFs: OSS Predictor. OSS TRFs for each participant calculated on the full dataset are shown. Note that TRFs are noisier than GT TRFs. Download Figure 1-4, TIF file. [file eneuro-11-ENEURO.0135-24.2024-s004.tif]

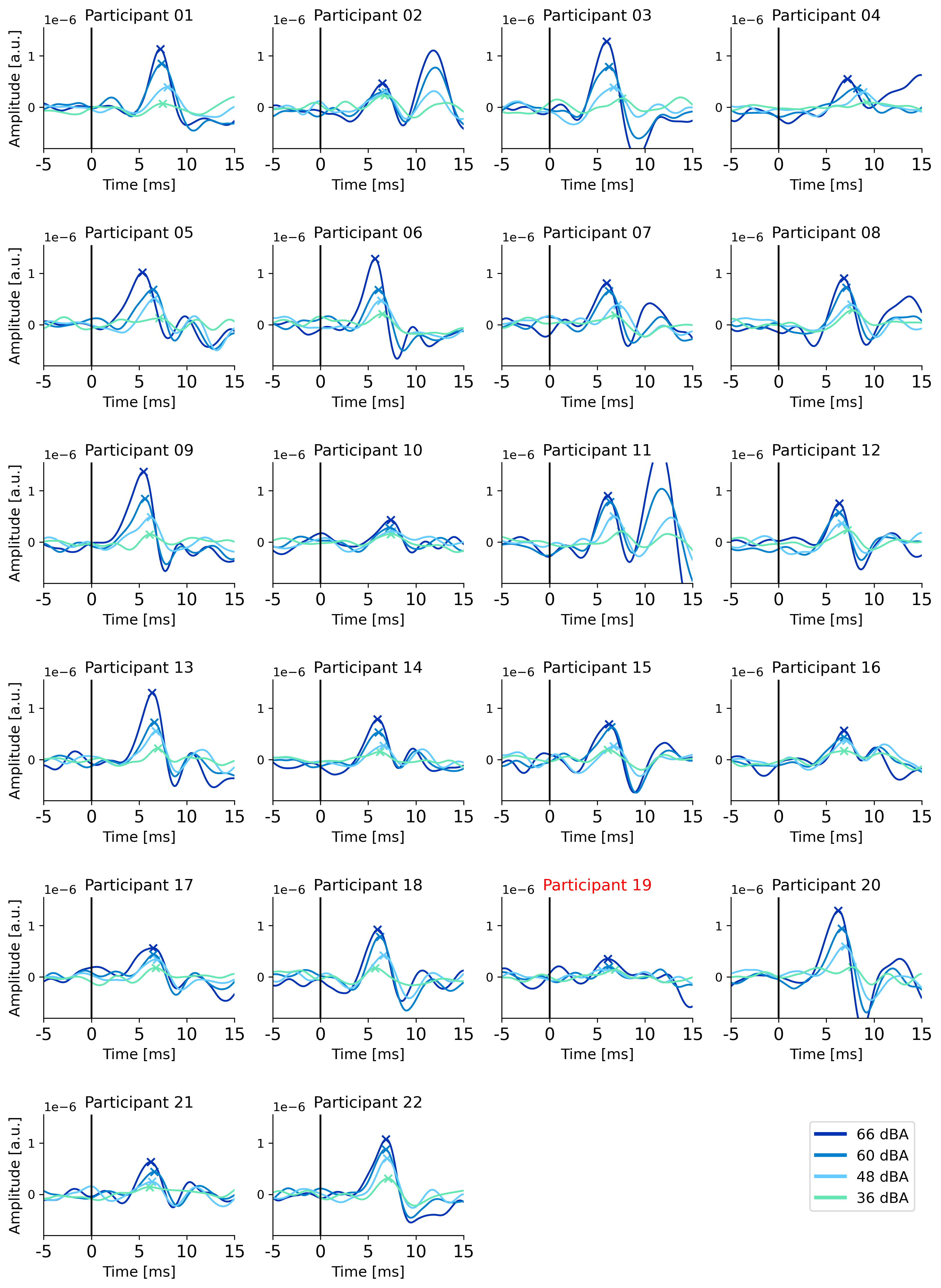

Supplement: Figure 1-5 — Individual Speech TRFs: OSSA Predictor. OSSA TRFs for each participant calculated on the full dataset are shown. Note that level-dependent latency effects are not as prominent. Download Figure 1-5, TIF file. [file eneuro-11-ENEURO.0135-24.2024-s005.tif]

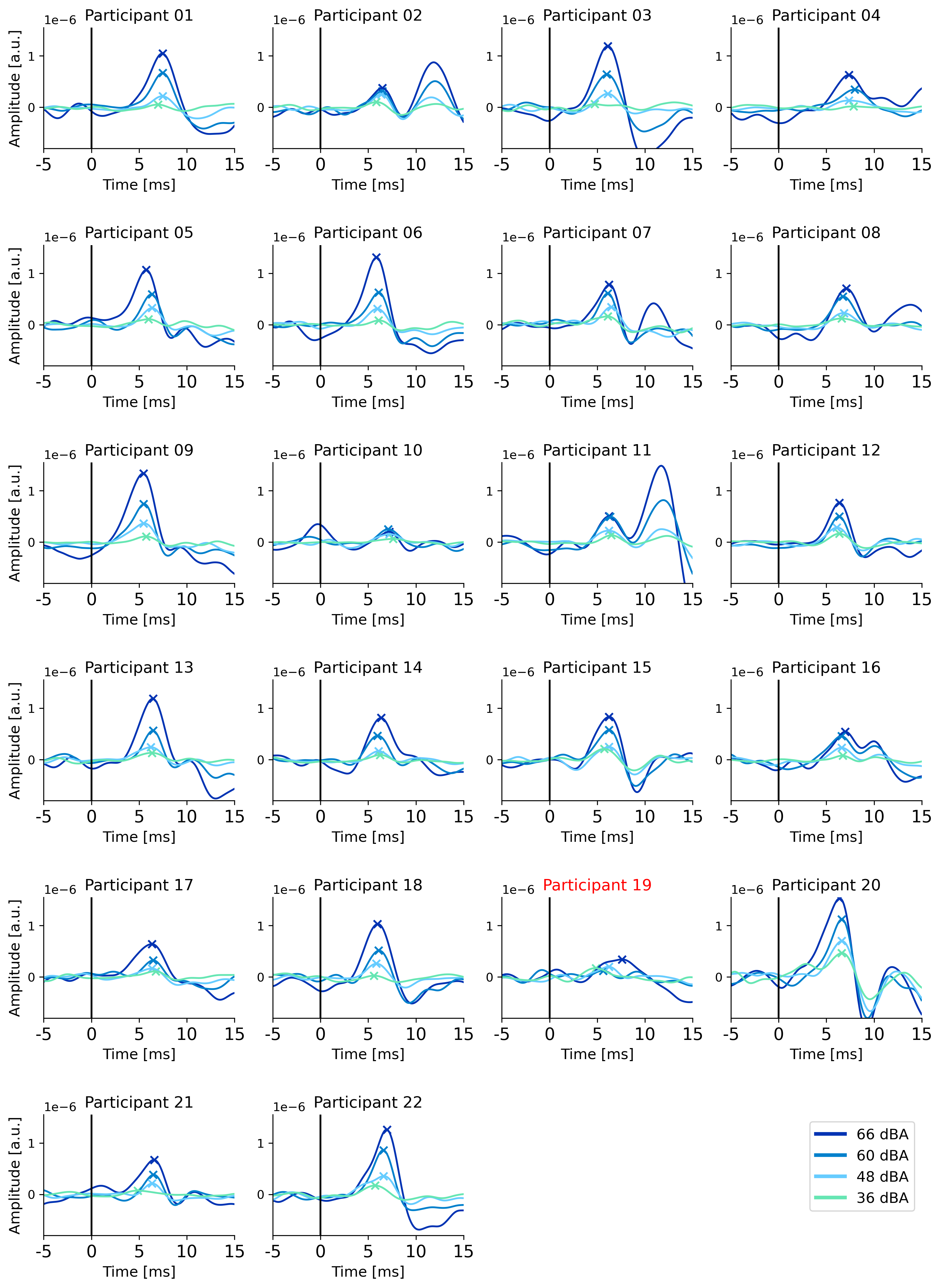

Supplement: Figure 1-6 — Individual Speech TRFs: ZIL Predictor. ZIL TRFs for each participant calculated on the full dataset are shown. Note that level-dependent latency effects are absent or against the expected trend in most participants. Download Figure 1-6, TIF file. [file eneuro-11-ENEURO.0135-24.2024-s006.tif]

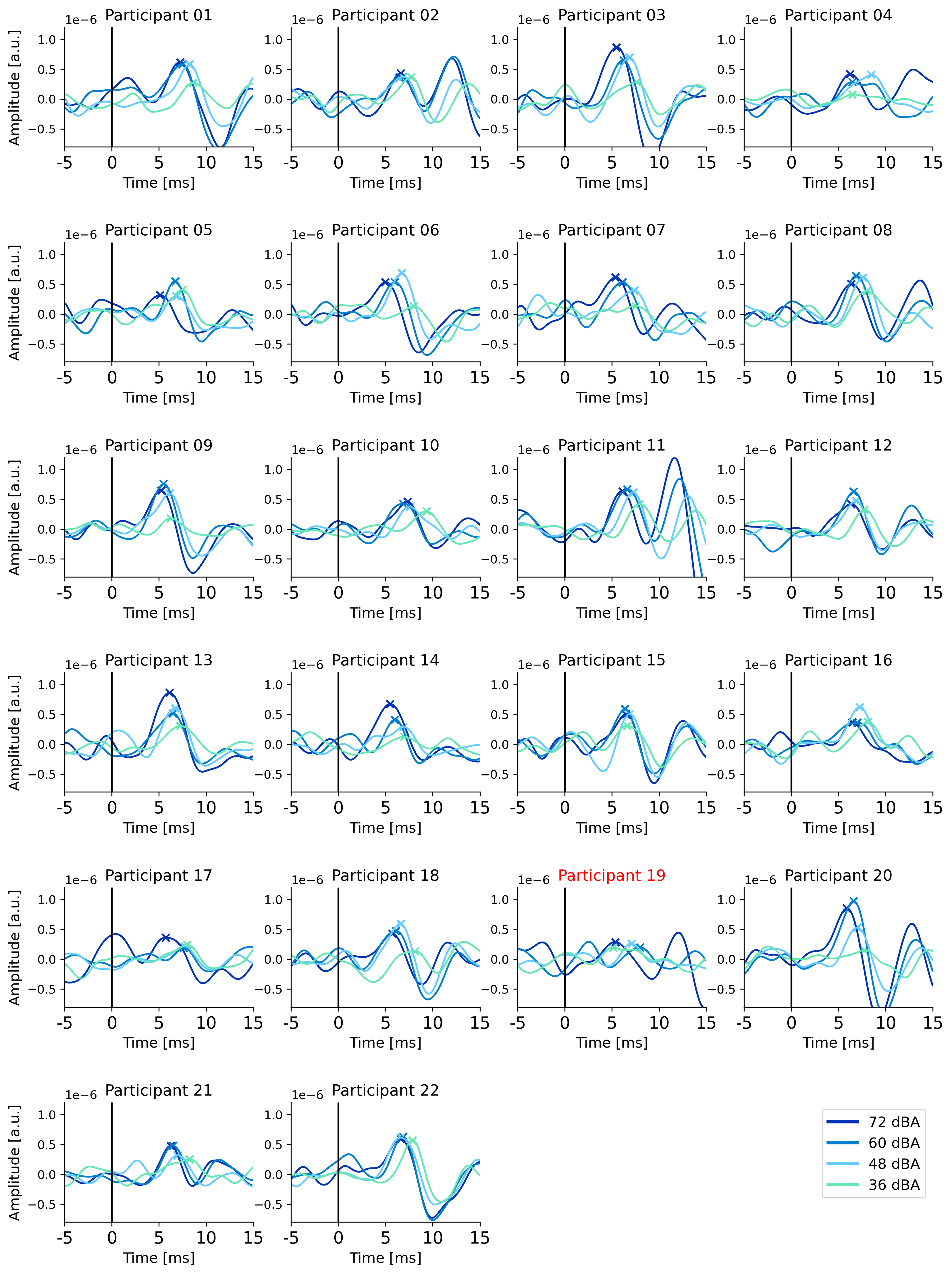

Supplement: Figure 4-1 — Individual Speech GT TRFs for the long duration fixed intensity condition. GT TRFs for each participant calculated on the 40 minutes of long duration fixed intensity condition are shown. Download Figure 4-1, TIF file. [file eneuro-11-ENEURO.0135-24.2024-s007.tif]

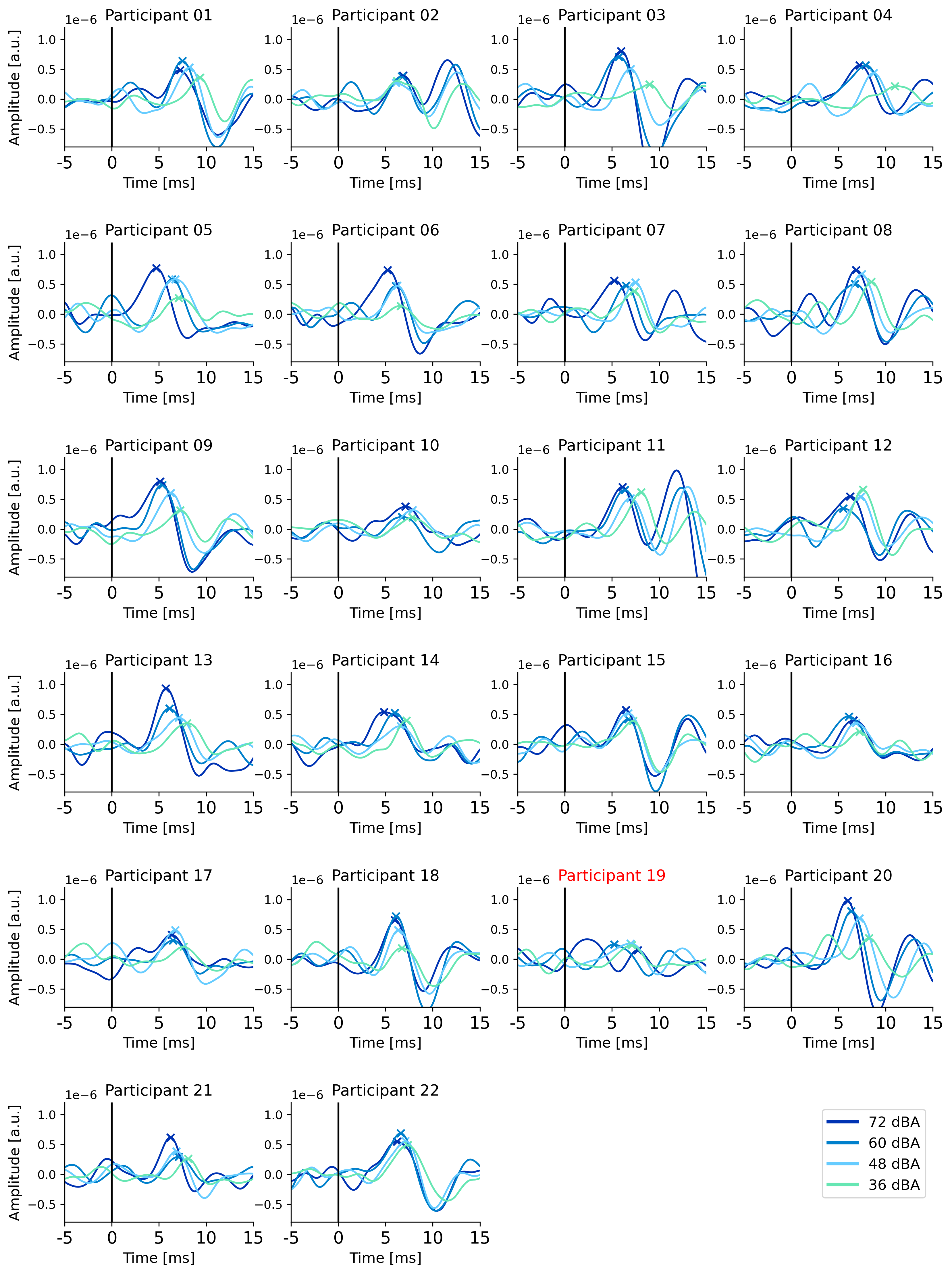

Supplement: Figure 4-2 — Individual Speech GT TRFs for the short duration fixed intensity condition. GT TRFs for each participant calculated on the 40 minutes of short duration fixed intensity condition are shown. Download Figure 4-2, TIF file. [file eneuro-11-ENEURO.0135-24.2024-s008.tif]

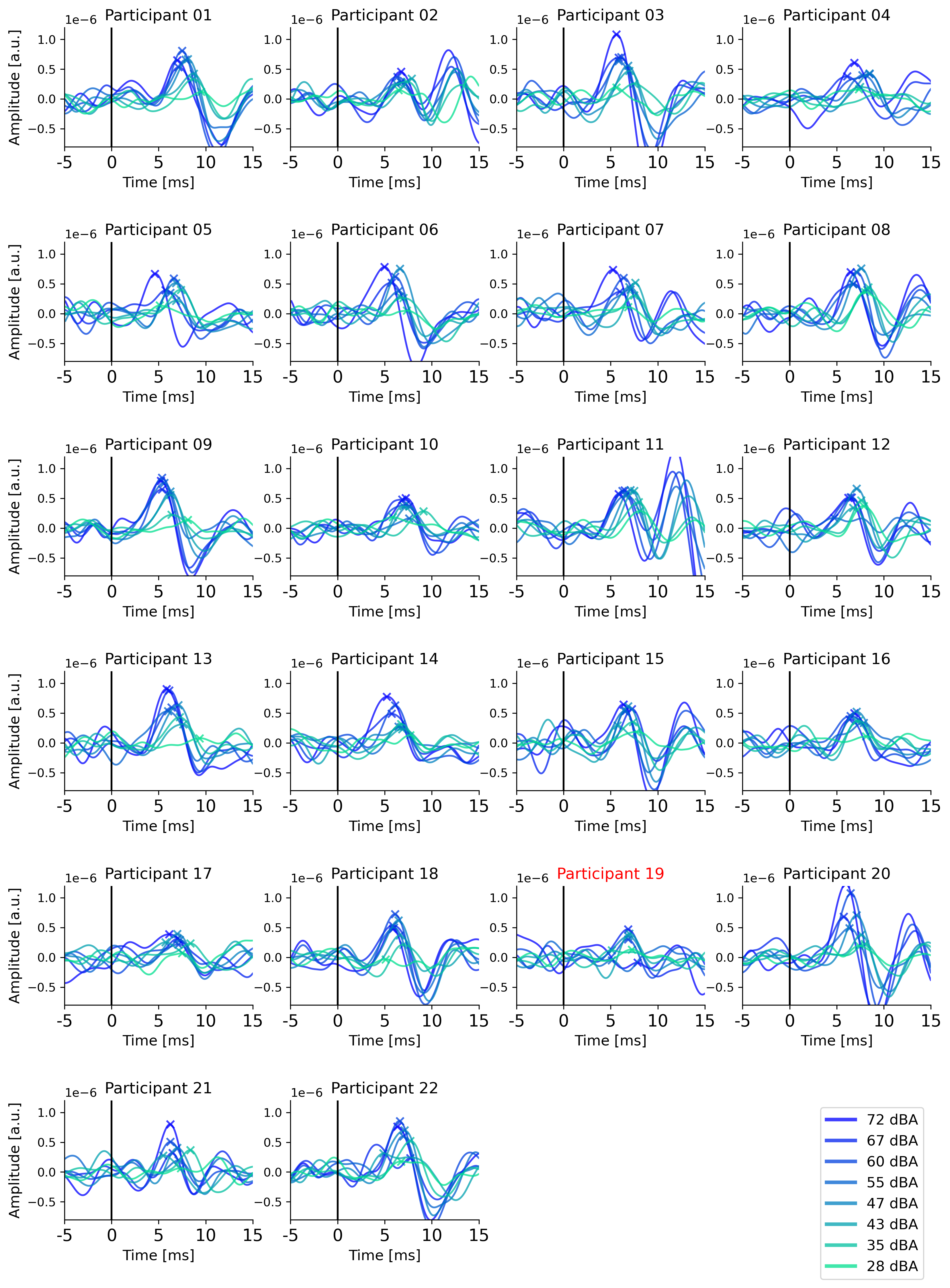

Supplement: Figure 6-1 — Individual Speech GT TRFs based on inherent changes in intensity level. GT TRFs for each participant calculated for the inherent level changes are shown. Note that most participants have a clear trend of increasing latency with decreasing level. Download Figure 6-1, TIF file. [file eneuro-11-ENEURO.0135-24.2024-s009.tif]
